# Red blood cell distribution width and Charlson comorbidity index help to identify frail polytraumatized patients: Experiences from a level I trauma center

**DOI:** 10.1007/s00508-022-02063-6

**Published:** 2022-08-09

**Authors:** Valerie Weihs, Stephan Frenzel, Michél Dedeyan, Thomas Heinz, Stefan Hajdu, Martin Frossard

**Affiliations:** Department of Orthopedics and Trauma Surgery, Medical University of Vienna, Währinger Gürtel 18–20, 1090 Vienna, Austria

**Keywords:** Polytrauma, Prognostic factors, Comorbidities, Anisocytosis, Concomitant disease

## Abstract

**Introduction:**

Little is known about the potential impact of the red blood cell distribution width (RDW) and pre-existing comorbidities on the late-phase survival of polytraumatized patients.

**Methods:**

A total of 173 polytraumatized patients were included retrospectively in this cohort study in a level I trauma center from January 2012 to December 2015. The Charlson comorbidity index (CCI) scores and RDW values were evaluated.

**Results:**

Out of all polytraumatized patients (*n* = 173), 72.8% (*n* = 126) were male, the mean ISS was 31.7 points (range 17–75) and the mean age was 45.1 years (range 18–93 years). Significantly higher RDW values (13.90 vs. 13.37; *p* = 0.006) and higher CCI scores (3.38 vs. 0.49; *p* < 0.001) were seen in elderly polytraumatized patients (age > 55 years). RDW values > 13.75% (*p* = 0.033) and CCI scores > 2 points (*p* = 0.001) were found to have a significant influence on the late-phase survival of polytraumatized patients. Age > 55 years (*p* = 0.009, HR 0.312; 95% confidence interval (CI) 0.130–0.749) and the presence of severe traumatic brain injury (TBI) (*p* = 0.007; HR 0.185; 95% CI 0.054–0.635) remained as independent prognostic factors on the late-phase survival after multivariate analysis.

**Conclusion:**

Even younger elderly polytraumatized patients (> 55 years of age) showed significant higher RDW values and higher CCI scores. In addition to the presence of severe TBI and age > 55 years, RDW value > 13.75% on admission and CCI score > 2 might help to identify the “younger” frail polytraumatized patient at risk.

## Key points

- Significantly higher RDW values on admission and significantly higher CCI scores were seen in the “younger” aging group (> 55 years of age) of polytraumatized patients.
- Our results suggest an association between the RDW values and rising age.
- RDW value > 13.75% on admission and CCI score > 2 might be implemented in risk stratification scores to identify the “younger” frail polytraumatized patient at risk.

## Background

Little is known about the impact of red blood cell distribution width (RDW) values on admission and Charlson Comorbidity Index (CCI) scores in polytraumatized patients, especially regarding the late-phase survival. A recent study showed that even so-called younger aging (> 55 years of age) polytraumatized patients had a higher late-phase mortality compared to younger ones [1]. Our study aims to identify additional potential prognostic factors that might help to identify the younger frail polytraumatized patient.

The RDW is a simple and inexpensive parameter which measures the variation in the size of red blood cells reflecting the degree of heterogeneity of erythrocyte volume (anisocytosis) [2]. Anisocytosis is common in chronic diseases [3–8]. An increase in RDW values has been described to predict mortality in patients with Fournier’s gangrene [9], severe sepsis or septic shock [10, 11] and is an independent predictor of all-cause mortality in patients after cardiac arrest [12] and in patients with acute heart failure [13]. Recent studies have reported increased RDW values in trauma patients as independent predictors of short-term and long-term mortality [14–18] but not in polytraumatized patients [19, 20]. Increased RDW levels in polytraumatized patients might play a prognostic role in patients with chronic diseases [20] and have shown a correlation with the probability to develop sepsis in trauma patients [19]. Significantly increased RDW values were observed in trauma patients especially in patients with head trauma [17, 21]. In patients with acute traumatic brain injury (TBI) the ratio of RDW to platelet count (RPC) was identified as predictor for mortality [17]. Higher RDW values were independent risk predictors of short-term and long-term hip fracture mortality [15, 22], especially in older patients with acute hip fractures [18]. In ICU patients, higher ward mortality was found at each quartile of RDW [23]. Furthermore, increased RDW values on admission are associated with higher in-hospital mortality in older patients [24].

The age-adjusted CCI is a well validated combined age-comorbidity score for prediction of the survival of patients based on cumulative patient comorbidities [25]. Pre-existing comorbidities have been shown to have a high impact on the functional outcome and mortality rates in trauma patients with hip fractures [26]. In trauma patients, higher CCI scores are associated with adverse events during the hospital stay [27–29] with increased hospital length of stay [30, 31] and increased risk for readmission after trauma surgery [28]. After survival of the acute phase of the trauma, the main limiting factors for late-phase survival in polytraumatized patients remain age > 55 years and the presence of a severe traumatic brain injury (TBI) [1]. RDW values on admission and CCI scores might be additional prognostic factors and help to identify the polytraumatized patient under risk for late-phase mortality.

Our study addresses this issue and investigates the incidence of pre-existing comorbidities in polytraumatized patients and the association between these comorbidities and RDW values on admission as we try to answer following questions:

- Is there an association between underlying comorbidities and the RDW values on admission in polytraumatized patients?
- Do polytraumatized patients present with higher RDW values independent from age or coexisting comorbidities?
- Is there a cut-off value for the RDW or the CCI score to predict the late-phase mortality in polytraumatized patients?

## Methods

The local Ethics Committee approved the study protocol. The study was conducted according to the principles expressed in the Declaration of Helsinki. In this study, 337 consecutive patients were retrospectively enrolled who were admitted to our hospital with severe injuries from January 2012 to December 2015.

Inclusion criteria for our analysis were: patients with an ISS > 16 points, an AIS > 3 points at least in 1 body region and at least 2 or more different body regions affected. For further analyses of the injury severity, patients were classified according to the new Berlin definition [32].

Patients with an isolated traumatic brain injury (TBI) (*n* = 101), patients with minor injuries (AIS < 3 points or ISS < 17 points) (*n* = 45) and patients younger than 18 years of age (*n* = 18) were excluded from this study, leading to 173 remaining included patients.

### Patient population

The baseline characteristics such as gender, age and clinical symptoms, the treatment protocols, the trauma mechanism as well as the short-term outcomes were reported. Possible prognostic factors such as severe traumatic brain injury (TBI) (AIS ≥ 3 points), age, the injury severity and RDW levels on admission were detected. Blood analysis including RDW levels (referred range 11.0–16.0%) was performed at the time of the patient’s arrival to the department of trauma surgery. A recent study from our study group identified age > 55 years as an independent predictor of late-phase survival in polytraumatized patients [1], therefore we decided to analyze our patient cohort according to this previously documented cut-off value: polytraumatized patients < 55 years of age and polytraumatized patients > 55 years of age. Three time-dependent events were defined: acute-phase death (within the first 24 h after the trauma), late-phase death (> 24 h within the hospital stay) and overall death (death at any time after the trauma).

### Comorbidity assessment

Pre-existing comorbidities and the age-adjusted CCI score were evaluated. Data records of all polytraumatized patients were screened for pre-existing comorbidities. The comorbidity assessment was performed using the CCI score including 19 different medical conditions and each comorbid condition ranges from 1 to 6 points to sum up the index score [25].

### Statistical analysis

The analysis of data focused on the relationship between RDW levels and concomitant comorbidities in polytraumatized patients and their potential influence on the primary outcome. Continuous variables are presented as means and standard deviations or medians and interquartile ranges (IQR). Categorical variables are provided with percentages. Descriptive statistics were used for demographic variables and clinical characteristics. Trauma mechanisms, injury characteristics and severity of injuries (classified with the AIS score) were examined. For detection of associations between qualitative variables a χ^2^-test or Fisher exact test was performed. For comparison between categorical and continuous variables Student’s *t* test was calculated. A two-sided *P* value of less than 0.05 was considered to indicate statistical significance. The Kaplan-Meier method was used to provide survival estimates, which were assessed with a log-rank test. Univariate Cox regression analysis was performed for evaluation of potential prognostic factors on the late-phase survival. Age, severe TBI (AIS ≥ 3 points), RDW values on admission and CCI scores were included in the univariate Cox regression analysis as potential confounders. Only significant factors (*p* < 0.05) in the univariate analysis were entered into the multivariate analysis. Stepwise forward multivariate Cox regression analysis was performed for identification of outcome prognosticators. The overall survival was calculated from the date of trauma to the date of death. Patients who died of unrelated causes were considered to have been censored. For the primary analysis of RDW association with the secondary and overall survival, Cox regression analysis was performed to calculate hazard ratio (HR) and 95% confidence intervals (CI). A receiver operating characteristics (ROC) curve was used to determine the area under the curve from RDW values and CCI scores. All data manipulation and statistical analysis was performed using IBM SPSS Statistics (statistics software for Macintosh, version 25.0; IBM Corp., Armonk, NY, USA). Statistical significance was set at *p* < 0.05.

## Results

A total of 173 patients met the inclusion criteria with a mean age of 45 years (18–93). Mean ISS score was 32 points (18 to 75 points) and 72.8% were male. The mean RDW value was 13.5% and the mean CCI score was 1.3 (0–8). The characteristics of patients are shown in Table 1.

**Table 1:** Patient characteristics listed by age beyond 55 years

		Polytraumatized patients < 55 years of age (*n* = 126)	Polytraumatized patients > 55 years of age (*n* = 47)	*P* value
*Mean ISS*		32 (17–75)	30 (17–75)	0.430
*Severe TBI*		64 (50.8%)	30 (63.8%)	0.126
*Berlin definition*		67 (53.2%)	26 (55.3%)	0.801
*Acute-phase death*		19 (15.1%)	3 (6.4%)	0.127
*Late-phase death*		9 (7.1%)	12 (25.5%)	0.001*
*Overall mortality*		28 (22.2%)	15 (31.9%)	0.189
*Cause of death*	TBI	5 (17.9%)	8 (53.3%)	0.027*
	Trauma	19 (67.9%)	3 (20%)	–
	MOF	2 (7.1%)	0	–
	ARF	1 (3.6%)	1 (6.7%)	–
	PE	0	1 (6.7%)	–
	Sepsis	1 (3.6%)	0	–
	Pneumonia	0	1 (6.7%)	–
	Hypoxia ^a^	0	1 (6.7%)	–
*Charlson Comorbidity Index*		0.49 (0–7)	3.38 (1–8)	< 0.001*
*RDW mean*		13.37 (12.0–20.20)	13.9 (12.1–17.5)	0.006*

*TBI* traumatic brain injury, *MOF* multiple organ failure, *ARF* acute renal failure, *PE* pulmonary embolism, *RDW* red blood cell distribution width

^a^hypoxic brain damage after resuscitation in one patient

*statistically significant

Of the patients 94 (54.3%) presented with pre-existing comorbidities. Main comorbidities were cardiovascular disease (*n* = 28; 15.6%), psychiatric disease (*n* = 29; 16.8%), neurological disease (*n* = 14; 8.1%) and drug abuse (*n* = 14, 8.1%). Incidences of cardiovascular disease (*p* < 0.001), oncological disease (*p* = 0.030), renal disease (*p* = 0.025) and overall comorbidities (*p* = 0.010) were statistically significantly higher in elderly patients. In contrast, drug abuse was seen more often in younger patients (*p* = 0.079) (Table 2). There were no gender differences regarding the incidence of comorbidities except for autoimmunological diseases which were seen more often in women (*p* = 0.027).

**Table 2:** Comorbidities in polytrauma patients listed by age beyond 55 years

		Polytraumatized patients < 55 years of age (*n* = 126)	Polytraumatized patients > 55 years of age (*n* = 47)	*p* value
*Comorbidities*		61 (48.4%)	33 (70.2)	*0.010
*Charlson Comorbidity Index*		0.49 (0–7)	3.38 (1–8)	*< 0.001
*DM*		1 (0.8%)	1 (2.1%)	0.465
*Mild liver disease*		7 (5.6%)	2 (4.3%)	0.732
*Localized malignancy, lymphoma or leukemia*		1 (0.8%)	4 (8.5%)	*0.006
*Metastasis*		0	1 (2.1%)	–
*HIV*		2 (1.6%)	0	0.385
*Moderate to severe chronic kidney disease*		5 (4.0%)	6 (12.8%)	*0.035
*MI*		0	0	–
*Heart failure*		2 (1.6%)	5 (10.6%)	*0.023
*COPD*		2 (1.6%)	2 (4.3%)	0.487
*Stroke*		1 (0.8%)	1 (2.1%)	0.465
*Dementia*		0	1 (2.1%)	0.101
*Hemiplegia*		1 (0.8%)	1 (2.1%)	0.465
*Connective tissue damage*		1 (0.8%)	1 (2.1%)	0.465
*Peptic ulcer disease*		2 (1.6%)	2 (4.3%)	0.299
*Peripheral vascular disease*		1 (0.8%)	1 (2.1%)	0.465
*Depression*		5 (4.0%)	5 (10.6%)	0.094
*Cardiovascular disease*		9 (7.1%)	18 (38.3%)	*< 0.001
*Drug abuse*		13 (10.3%)	1 (2.1%)	0.079
*Psychiatric disease*		21 (16.7%)	8 (17%)	0.956

*DM* diabetes mellitus, *HIV* human immunodeficiency virus, *MI* myocardial infarction, *COPD* chronic obstructive pulmonary disease

*statistically significant

**Fig. 1:**
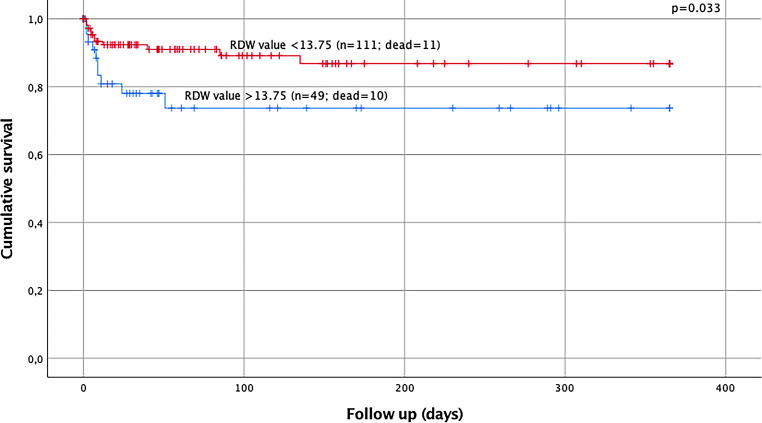
Kaplan Meier curve analysis for RDW values on admission and the influence of the RDW value > 13.75% on admission on the late-phase survival of polytrauma patients

Mean RDW value was 13.52% (range 12.00–20.20%). No gender differences in RDW values could be detected. Neither patients with higher ISS scores, nor patients having attempted suicide, nor patients with severe TBI showed different RDW levels on admission. Without any statistical significance, higher RDW values were seen in patients who died in the late phase of the trauma (13.74 vs. 13.49; *p* = 0.320) and in patients with pre-existing comorbidities (13.66 vs. 13.34; *p* = 0.066). In contrast, elderly patients presented with statistically significant higher RDW levels on admission (13.90 vs. 13.37; *p* = 0.006). The RDW value showed a statistically significant correlation with patient age (r = 0.278; *p* < 0.001). Based on a ROC analysis the Youden index suggests an optimal RDW cut-off value of 13.75% on admission to predict late-phase mortality. The AUC for predicting late-phase mortality using the RDW value on admission was 0.571 (*p* = 0.292). Survival analysis was calculated for late-phase mortality based on RDW values on admission. Results showed that a RDW value > 13.75% was a predictor for late-phase mortality (*p* = 0.033) (Fig. 1).

**Fig. 2:**
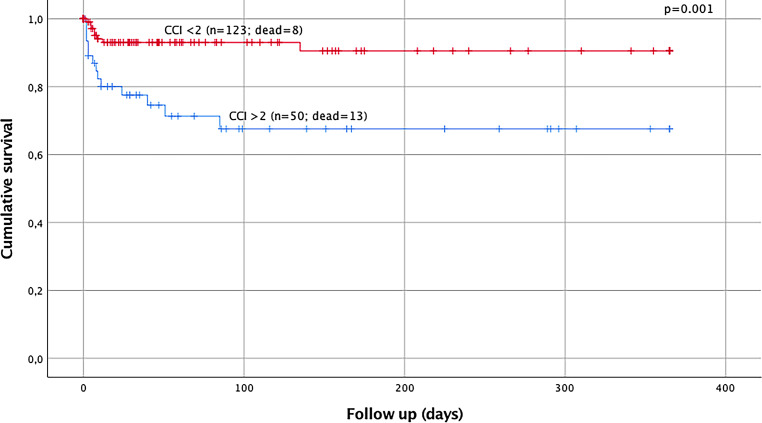
Kaplan Meier curve analysis for Charlson Comorbidity Indices (CCI) and the influence of the CCI score > 2 points on the late-phase survival of polytrauma patients

Mean CCI score in the overall cohort was 1.28 (range 0–8). Elderly patients showed statistically significant higher CCI scores (3.38 vs. 0.49; *p* < 0.001). Additionally, the CCI score was significantly higher in non-suicidal patients (0.38 vs. 1.42; *p* = 0.014) reflecting the younger age of suicidal patients. Higher CCI was also detected in patients classified according to the new Berlin definition (1.54 vs. 0.98; *p* = 0.058) and in patients with severe TBI (1.51 vs. 1.00; *p* = 0.085) without any statistical difference. The CCI score showed a statistically significant correlation with patien age (r = 0.771; *p* < 0.001). Statistically significant higher CCI scores were also detected in patients who died in the late phase of the trauma (2.62 vs. 1.10; *p* = 0.001). Based on a ROC analysis, the Youden index suggests an optimal CCI score cut-off value of 2 points on admission to predict late-phase mortality. The AUC for predicting late-phase mortality using the CCI score on admission was 0.685 (*p* = 0.006). Survival analysis was calculated for late-phase mortality based on CCI values on admission. Results of the log-rank test (*p* = 0.001) showed that this CCI value was a predictor of late-phase mortality (Fig. 2). After multivariate Cox regression analysis, severe TBI (*p* = 0.007, HR 0.175; 95% CI 0.054–0.635) and age > 55 years (*p* = 0.009; HR 0.312; 95% CI 0.130–0.749) were identified as independent negative prognostic factors for the late-phase survival (Table 3).

**Table 3:** Univariate and multivariate Cox regression analysis

	Univariate Cox regression			Multivariate Cox regression		
	*p* value	HR	CI	*p* value	HR	CI
Age > 55 years	0.002*	0.257	0.108–0.611	0.009*	0.312	0.130–0.749
*Severe TBI*	0.003*	0.159	0.047–0.539	0.007*	0.185	0.054–0.635
*CCI >* *2*	0.002*	0.249	β.103–0.602	n. s.	–	–
*RDW >* *13.75*	0.040*	0.407	0.173–0.960	n. s.	–	–

*TBI* traumatic brain injury, *CCI* Charlson Comorbidity Index, *RDW* red blood cell distribution width

*statistically significant

## Discussion

Our study contributes several insights regarding the late-phase survival of polytraumatized patients and possible prognostic factors that might help in the identification of the frail polytraumatized patient at risk.

Firstly, we have found a tendency towards higher RDW values on admission in our cohort of patients with pre-existing comorbidities. Although no prognostic value of RDW on admission in trauma patients has been shown by Kong et al. with respect to the 28-day mortality, higher RDW values in the acute phase of the trauma were found to be strong independent predictors of short-term mortality [14]. RDW has also been associated with a graded risk for the 30-day and 1‑year mortality especially in older trauma patients and elderly patients with acute hip fractures [16, 18]. In our cohort of patients higher RDW values on admission were seen especially in the cohort of patients who died within the late phase of the trauma. Additionally, significantly higher RDW values on admission in the group of polytraumatized patients > 55 years of age were documented. RDW values might have a prognostic role in trauma patients with chronic diseases [20], higher RDW values on admission might be able to add further valuable information. Neither patients with higher ISS nor patients with severe TBI showed differences in the RDW values on admission. An association between the RDW values and rising age could be seen in our analysis. Accordingly, higher RDW values on admission were also documented in patients who died within the late phase of the trauma. Therefore, RDW might be included in the risk stratification of polytraumatized patients who survived the acute phase of the trauma.

Secondly, we were able to demonstrate that significantly higher CCI scores were seen in aging polytraumatized patients and in patients who died within the late phase. Higher CCI scores were found to be significantly associated with increased hospital length of stay, an increased risk for readmission and increased risk for in-hospital mortality [30, 31, 33–35]. According to our data, polytraumatized patients with pre-existing health conditions showed higher RDW values. In daily clinical practice, a detailed evaluation of concomitant health conditions in polytraumatized patients is often not feasible. Therefore, the assessment of RDW might help to identify the frail polytraumatized patient at risk.

Thirdly, we were able to demonstrate that RDW values on admission > 13.75% and CCI scores > 2 had a significant influence on the late-phase mortality of polytraumatized patients. An optimal cut-off value for the RDW > 14.4% on day 1 and > 14.7% for the 28-day all-cause mortality has been suggested by Kong et al.; however, patients with underlying malignancies or autoimmune diseases were excluded from their study population [14]. As suggested by others [14–16, 21], RDW as a simple and inexpensive biomarker might be integrated into validated diagnostic algorithms.

Summarizing, our study was able to show that even “younger” aging polytraumatized patients present with higher RDW values and higher CCI scores reflecting underlying concomitant comorbidities and that both values have an influence on late-phase survival of these patients.

## Limitations and strengths

This retrospective non-randomized, single-center study has the characteristic limitations of registry data and post hoc analyses. Potential limitations of this analysis include the small sample size; however, the analysis of data especially regarding the underlying comorbidities and other outcome parameters and their association with the late-phase mortality have been evaluated with high accuracy. Furthermore, although a tendency towards higher RDW values in patients with pre-existing comorbidities could be seen, further studies with higher numbers of patients are needed to underline this difference. An inherent selection bias might be present due to the retrospective design of this study. Specific strengths and a sign of quality of this study are the careful analysis of data in all consecutively included patients.

## Conclusion

In conclusion, even younger aging polytraumatized patients (> 55 years of age) showed significantly higher RDW values on admission and higher CCI scores. In addition to the presence of severe TBI and age > 55 years, RDW value > 13.75% on admission and CCI score > 2 might be implemented in risk stratification systems to identify the “younger” frail polytraumatized patient at risk.
